# Glutamatergic transmission regulates locomotory behavior on a food patch in *C. elegans*

**DOI:** 10.17912/micropub.biology.000332

**Published:** 2020-11-28

**Authors:** Trevor Wolf, Ariana Perez, Gareth Harris

**Affiliations:** 1 Biology Program, 1 University Drive, California State University Channel Islands, Camarillo, Ca, 93012

**Figure 1. Glutamatergic signaling regulates worms staying on a food patch:**
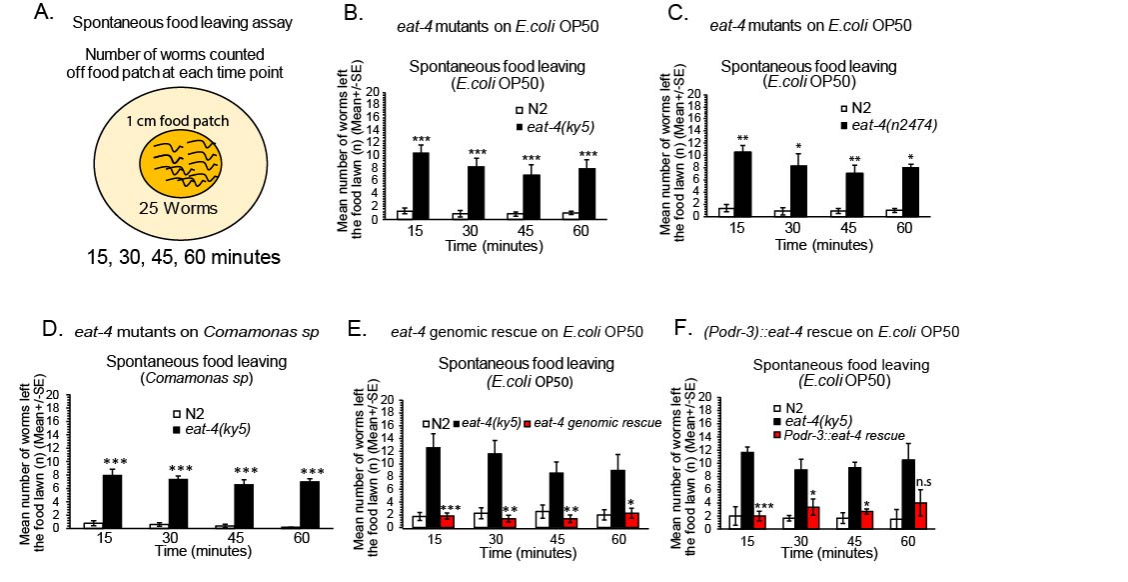
**A-F** **A)** Schematic of spontaneous food patch leaving assay (See methods), **B)** *eat-4(ky5)* mutants examined for spontaneous food leaving on an *E.coli* OP50 food patch (n=5), *eat-4(ky5)* mutants are compared to wild type N2 animals tested in parallel, **C)** *eat-4(n2474)* mutants were examined for spontaneous food leaving on a *E.coli* OP50 food patch (n=4), *eat-4(n2474)* mutants were compared to wild type N2 animals tested in parallel, **D)** *eat-4(ky5)* mutants were examined for spontaneous food leaving on a *Comamonas sp* food patch (n=4), *eat-4(ky5)* mutants were compared to wild type N2 animals tested in parallel, **E)** Full-length *eat-4* genomic rescue was performed for spontaneous food leaving on an *E.coli* OP50 food patch (*eat-4* genomic rescue was compared to *eat-4(ky5)* mutants for spontaneous food leaving), (n=5), **F)** *eat-4* rescue in *odr-3* expressing neurons was performed for spontaneous food leaving on a *E.coli* OP50 food patch (in *eat-4(ky5)* mutants) (n=3), selective *eat-4* rescue in *odr-3* expressing neurons were compared to *eat-4(ky5)* mutants tested in parallel. Both wild type and *eat-4* mutants were examined in parallel to rescue animals. For all rescue experiments in Fig. 1E and Fig. 1F, the rescue strains were compared to *eat-4(ky5)* mutant animals. For all data analysis, a *student’s t test* was performed when comparing wild type animals to all mutant animals tested on the same day in parallel conditions. Mean ± SEM, Student’s *t-t*est, * p ≤ 0.05, p ≤ 0.01**, p ≤ 0.001***. For all assays n= number of days tested. n.s=not statistically significant, White bars=N2, Black bars=mutants, Red bars=rescue.

## Description

Sensation of environmental cues and decisions made as a result of processing of specific sensory cues underlies a myriad of behavioral responses that control every-day life decisions and ultimately survival in many organisms. Despite the appreciation that organisms can sense, process, and translate sensory cues into a behavioral response, the neural mechanisms and molecules that mediate these behaviors are still unclear. Neurotransmitters, such as glutamate, have been implicated in a variety of sensory-dependent behavioral responses, including olfaction, nociception, mechanosensation, and gustation (Mugnaini *et al.*., 1984, Wendy *et al.*., 2013, Daghfous *et al.*., 2018). Despite understanding the importance of glutamate signaling in sensation and translation of contextual cues on behavior, the molecular mechanisms underlying how glutamatergic transmission influences sensory behavior is not fully understood. The nematode, *C. elegans*, is able to sense a variety of sensory cues. These types of sensory-dependent behavioral responses are mediated through olfactory, gustatory, mechanosensory and aerotactic circuits of the worm (Lans and Jansen, 2004, Milward **et al.*,* 2011, Bretscher *et al.*., 2011, Kodama-Namba *et al.*., 2013, Ghosh **et al.*,* 2017). Odor guided behavior toward attractants, such as, food cues requires neurotransmitters, that include, glutamate (Chalasani *et al.*., 2007, Chalasani *et al.*., 2010). More specifically, once on a food source, wild type N2 hermaphrodites will generally be retained on a food source (Shtonda and Avery, 2006, Milward *et al.*., 2011, Harris *et al.*., 2019). The types, quality, pathogenicity, and perception of food can modulate food recognition, food leaving rates, and overall navigational strategies towards food (Zhang *et al.*., 2005, Shtonda and Avery, 2006; Ollofsson *et al.*., 2014). These types of behaviors are based on detection of environmental cues, including oxygen, metabolites, pheromones, and odors. Food leaving behaviors have been shown to be influenced by a number of neuronal signals (Shtonda and Avery, 2006, Bendesky *et al.*., 2011, Ollofsson *et al.*., 2014, Meisel *et al.*., 2014, Hao *et al.*., 2018).

In this present study using our food patch behavioral paradigm, we examined the importance of glutamatergic transmission in the ability of worms to stay on a small food patch. This study has identified a role for glutamatergic transmission in keeping well-fed worms on a small food patch.

We have examined neurotransmitter systems and their role in the regulation of spontaneous food leaving, while residing on a food patch. Previous studies have implicated molecules in food recognition, dwelling and spontaneous food leaving (Shtonda and Avery, 2006, Bendesky *et al.*., 2011, Busch and Olloffson, 2012, Olofsson *et al.*, 2014). We first examined wild type N2 food leaving after worms were transferred to a small *E.coli* OP50 food patch (15, 30, 45 and 60 minutes after transfer to a food patch) (Fig. 1A-1B). Wild type animals generally leave an *E.coli* OP50 food patch across 0 – 60 minutes infrequently, approximately 3 out of 20-25 worms left an *E. coli* OP50 food patch across at least the first 1 hour (Fig. 1A, Schematic of behavior assay). We wanted to examine other bacterial food sources in this experiment to assess spontaneous food leaving. We did not choose bacterial species, such as, *Pseudomonas aeruginosa* (PA14), due to previous studies showing significant food leaving in wild type worms incubated on these types of pathogenic bacteria food lawns over time (Meisel *et al.*., 2014). We tested wild type N2 worms on an additional food patch, *Comamonas sp* (Fig. 1D). Wild type animals left with similar rates when compared to *E. coli* OP50 (Fig. 1B, and 1D). Food leaving rates taken together suggest that wild type N2 worms rarely leave a food patch which agrees with previous appreciation of wild type N2 animals showing low numbers of *E. coli* OP50 food leaving across the first hour after transfer of wild type N2 worms to a food patch (Harris *et al.*., 2019). We then sought to investigate the neural mechanisms that mediate staying on a food patch during the first hour.

We examined glutamatergic signaling in this food patch behavior through examination of *eat-4* mutants. *eat-4* encodes a vesicular glutamate transporter in *C. elegans* (Lee *et al.*., 1999). Interestingly, *eat-4* mutants were significantly enhanced food leavers (*eat-4(ky5*) (Fig. 1B, See time course over 60 minutes, Lee *et al.*., 1999). *eat-4(ky5)* mutants show a significant increase in the number of worms that were not on the food at time points across 15, 30, 45 and 60 minutes (Fig. 1B). This could be repeated by testing a second allele of *eat-4* with significantly reduced function, *eat-4(n2474)* (Fig. 1C, Lee *et al.*., 1999). Suggesting, *eat-4* removal disrupts normal food patch behavior on an *E. coli* OP50 food patch. To confirm this phenotype in *eat-4* mutants, we examined *eat-4* mutants that were rescued with a full-length genomic fragment (Rankin *et al.*., 2000; Fig. 1E). *eat-4* mutants were rescued for food patch behavior (Fig. 1E), confirming that *eat-4*, and thus glutamatergic signaling is important in this behavior. To examine whether this *eat-4*-dependent phenotype can be observed on more than one food type, we examined *eat-4* mutants on additional food types. We examined *eat-4* mutants for their spontaneous food leaving on an *Comamonas sp food* patchto see if this food type also fails to keep *eat-4* mutants on the food patch over 1 hour (Fig. 1D). *eat-4* mutants again did show an obvious food leaving phenotype when compared to wild type animals on *Comamonas sp* food lawns (*eat-4(ky5)* tested), Fig. 1D). Suggesting, that changing the type of food patch, does not significantly reduce *eat-4* mutant-dependent increase in spontaneous food leaving on the food patch (Fig. 1). Therefore, *eat-4* mutants fail to stay on multiple types of food, including *E. coli* OP50 and *Comamonas sp*. To determine where glutamate signaling is important for keeping worms on a food patch, we examined the site of *eat-4* in mediating staying on a food patch. We examined strains that selectively rescue *eat-4* in specific sets of glutamatergic neurons. *eat-4* is expressed in over 30 neurons in the hermaphrodite, including, sensory and interneurons (Serrano-Saiz *et al.*., 2013). We examined *eat-4* mutants that were rescued using the *odr-3* promoter that expresses strongly in the AWC sensory neurons, weakly in the AWB sensory neurons and faintly in the AWA, ASH and ADF sensory neurons (Royaie *et al.*., 1998, Chalasani *et al.*., 2007, Calhoun *et al.*., 2015, Fig. 1F). Interestingly, *eat-4* rescue using an *odr-3* promoter rescued *eat-4(ky5)* mutants for staying on a food patch (Fig. 1F). This overall study shows that glutamatergic transmission is important in keeping worms on a food source.

## Methods

**Worm, media, plate preparation and maintenance.** All wild type animals were grown at 20-22 degrees with sufficient *E.coli* OP50 food source prior to examination of all worms (Brenner **et al.*,*1974). All wild type worms tested in this present study were 1 day old adult hermaphrodite animals under non-starved conditions. To prepare the food lawn used in the assay, NGM-bacterial culture was prepared the night before the assay (40 mL + *E.coli* OP50 colony) and then 55 µL of the overnight culture was added to the center of a 6 cm NGM plate and left to dry for 2 hours to produce a 1 cm food lawn. After 2 hours, worms were added to the food patch to examine food leaving across the next 60 minutes.

**Spontaneous food leaving on *E. coli* OP50 and *Comamonas sp*.** To determine if glutamate signaling is involved in the present behavior, we measured food leaving as follows: Wild type young adults were added to the relevant food lawn (1 cm diameter, Harris *et al.*., 2019). The number of worms found off the food were recorded every 15 minutes for 1 hour from the point of adding the worms to the food lawn (0 – 60 minute assay, see Schematic, Fig. 1A). We then compared mutant worms with wild type animals tested in parallel. 3-6 days were performed for each mutant or transgenic rescue lines tested when compared to wild type animals. For data analysis for *E.coli* OP50 or *Comamonas sp* food lawns, a *student’s* t test was performed to compare all mutants vs wild type (N2) animals, or mutant animals vs transgenic rescued animals. Mean ± SEM, Student’s *t-t*est, * p ≤ 0.05, **p ≤ 0.01, ***p ≤ 0.001.

## Reagents

**Strain list: All strains were purchased from CGC**

The following strains were examined in this experiment. MT6308 *eat-4(ky5)III*, MT6318 *eat-4(n2474)III, CX6827 kyEx844* contains [*odr-3::eat-4 + elt-2::GFP]*, *DA1242 eat-4(ky5) III; lin-15B&lin-15A(n765)X; adEx1242. E.coli* OP50 bacteria was purchased from CGC (Minnesota), *Comamonas sp* was purchased from CGC (Minnesota). Strains were provided by the CGC (Caenorhabditis Genetics Center), which is funded by NIH Office of Research Infrastructure Programs (P40 OD010440).
